# An atypical case of serotonin syndrome with normal dose of selective serotonin inhibitors: A case report

**DOI:** 10.1097/MD.0000000000015554

**Published:** 2019-05-13

**Authors:** Yun Liu, Hailong Yang, Fei He, Peng Xu, Hanwen Tong, Yao Liu, Jie Ni, Qiulin Zhang, Jun Wang

**Affiliations:** a Department of Emergency; b Department of Psychiatry, Nanjing Drum Tower Hospital, Affiliated Hospital of Nanjing University Medical School, Nanjing, China.

**Keywords:** citalopram, donepezil, NMS, olanzapine, P450 enzyme system, paroxetine, SS, SSRIs

## Abstract

**Rationale:**

As increasing frequency of serotonergic drug use, SS (serotonin syndrome) occurred more than ever. But clinicians have not enough knowledge and experience about SS as a potentially life-threatening condition. SS is usually caused by the increased serotonin activity in the central nervous system which may due to a serotonergic agent overdose or the concomitant use of 2 or more serotonergic antidepressants. We report a case of SS due to a normal dose of selective serotonin inhibitors (SSRIs) thus to remind clinicians to pay attention to such patients and make an early diagnosis and initiation of therapy in the clinical practice.

**Patient concerns:**

We report here a 49-year-old man presented with lethargic, less communicative, and insomnia for 20 days while a diagnosis of depression was considered and he was treated with SSRIs.

**Diagnosis:**

The patient in our case fulfilled the 3 criteria existed now for diagnosing SS, including the Sternbach criteria, Radomski revised diagnostic criteria, and the Hunter serotonin toxicity criteria.

**Interventions:**

All the antidepressants were stopped and cyproheptadine with an initial dose of 12 mg a day was started along with supportive care. The patient was also admitted to emergency intensive care unit for further treatment. He was sedated and paralyzed by intravenous Midazolam and Clonazepam along with physical cooling and supportive care.

**Outcomes:**

All of the patient's symptoms abated gradually and he soon could get off the bed and be communicative. Finally, the patient made a full recovery and he was discharged from the hospital.

**Lessons:**

Our case suggests an atypical clinical course while the medicine the patient takes was not in so much dose. We assumed that there may have been some variation in metabolism of these agents, resulting in increased possibility that led to the subsequent syndrome. Thus, it is essential for clinicians to keep in mind when patients taking serotonergic agents who demonstrate acute change in their mental status. Besides, clinicians should be aware of such patients who seem to be sensitive to SSRIs, who may require a genetic testing before the initiation of SSRI therapy.

## 1 Introduction

Serotonin syndrome (SS) is an iatrogenic, drug-induced clinical syndrome which may be potentially life-threatening as increasing frequency of serotonergic drug use occurred more than ever .^[1]^ SS is usually caused by the increased serotonin activity in the central nervous system which may due to a serotonergic agent overdose or the concomitant use of 2 or more serotonergic antidepressants .^[1–5]^ But few cases have reported the SS caused by normal-dose of serotonergic agent since it was first reported in 1960 by Oates and Sjoerdsma.^[6]^ SS is characterized by a triad symptom including autonomic instability (dilated unresponsive pupils, abdominal pain, profuse sweating, hyperthermia, tachycardia and flushing, etc.), neuromuscular hyperactivity (bilateral Babinski sign, bruxism. hyperreflexia, shivering and tremor, etc.), and mental status changes (agitation, hypomania, restless, disorientation, and confusion). The clinical manifestation of SS ranges from mild forms to fatal ones, patients with SS may not present with symptoms of all of the 3 categories mentioned above, while some patients may just show the symptom of increased neuromuscular excitability. Severe SS is medical emergency, thus, the early diagnosis and initiation of therapy is vital in the clinical practice. But the awareness of physicians, especially, of first-line physicians, is still inadequate, as 85% of physicians were unaware of SS as a clinical diagnosis.^[7]^

We present a case of SS caused by normal dose selective serotonin inhibitors (SSRIs), which fulfilled the Sternbachcriteria, Radomski's revised criteria and Hunter criteria. The patient in our case responded well to the withdraw of the relevant agents and the administration of cyproheptadine. In this case report, we also summarized the etiology, clinical presentation, diagnostic protocols, differential diagnosis of SS, and the gene polymorphisms involved in the metabolism of these drugs. We anticipate that our case report could provide more insight to clinicians to be aware of such syndrome.

## 2 Case report

A case of a 49-year-old man presented with lethargic, less communicative, and insomnia for 20 days was admitted into local hospital. The electroencephalogram showed a medium abnormality, while cranial computed tomography was normal. Therefore, wuling capsule, paroxetine (10 mg every day) were administrated to the patient, while 3 days later, the patient was presented with headache, nausea, and vomiting, then the medicine therapy was stopped and the patient was transferred to our hospital for further treatment on February 13, 2018. The patient had no history of drug or alcohol use, no medical history, and no drug allergies. The inpatient parameters were: body temperature 36.5°C, pulse 108 beats/min, blood pressure 153/97 mm Hg, and respiratory rate 20 breaths/min. On physical examination, no abnormalities were observed and neurological examination was normal. The results of cerebrospinal fluid (CSF) showed: the level of protein was 257.3 mg/L (normal range: 150–450 mg/L), glucose was 3.49 mmol/L (2.5–4.5 mmol/L), no white blood cell, and the culture of CSF was negative. Laboratory investigations including routine blood test revealed slightly increased white blood cell (9.6×10^9^/L, normal range: 0–8×10^9^/L). The thyroid function was normal. The manteaux test was negative. Examination and electroencephalogram were normal. Cranial magnetic resonance imaging was normal. ECG showed a sinus rhythm with a heart rate of 122 beats/min.

On admission, the body temperature of the patient reached to 37.8 °C, pulmonary computed tomography showed no signs of infection, so the diagnosis of encephalitis was suspected, and the empirical injectable acyclovir was started, along with 40mg methylprednisolone and CZX for antibiotic treatment. But the results of CSF were normal, thus the mental disorder cannot be excluded. Cipramil with a dose of 10 mg was started on 15th, Aricept with an initial dose of 5 mg was administrated on 15th, and Olanzapine with a starting dose of 2.5 mg was administrated on 18th to relieve the mental symptom but they did not work very well.

The symptom did not improve with consistent fever but no obvious infections were detected. The drug fever was considered while all intravenous medications were stopped on 20th, but the patient was still in hyperthermia and showed an increase in muscular tension. Sooner, the patient arose rigidity in extremities with trismus and shaking of 4 limbs, but he was still in consciousness and responsive. Due to the rigidity of extremities, a blood creatine phosphokinase (CPK) was checked on 24th, and it was 183 U/L (normal range: 55–170 U/L).

The symptom of the patient worsened day by day, he became more and more apathetic and exhibited a high fever (the heat peak reached to 39°C) coupled with photophobia, Estazolam was administrated to release symptom. After consulting psychiatry department, considering the medication history and clinical manifestation, the diagnosis of “serotonin syndrome” was considered. All the antidepressants were stopped on 22nd and Cyproheptadine with an initial dose of 12 mg a day was started along with supportive care. But the patient did not have any improvement in symptom but gradually accompanied by consciousness disturbance and further increased body temperature with diaphoresis and tachycardia. The patient was admitted to emergency intensive care unit for further treatment. He was sedated and paralyzed by intravenous Midazolam and Clonazepam along with physical cooling and supportive care. The patient was drowsy and appeared no more tremor and his body temperature decreased gradually. Oral Clonazepam was administrated 2 mg a day at first on 26th, then reduced to 1 mg a day, all of the patients’ symptom abated except slight fever and mild coma, then the patient was transferred to normal ward on February 28th. While on March 4th^,^ the patient could get off the bed and be communicative, while the Cyproheptadine was reduced to 6 mg a day and Clonazepam reduced to 0.5 mg per night. On 9th, the patient made a full recovery and he was discharged from the hospital. The written informed consent was obtained from the patient for publication of the case report.

## 3 Discussion

Antidepressants are frequently used nowadays while SSRIs and serotonin-noradrenaline (SNRIs) are the major drugs.^[8]^ Based on the French pharmacovigilance reports, SSRIs were involved in 42.1% of SS.^[9]^ The SS is one of the adverse events (AEs) of SSRIs and SNRIs which can be fatal. The concomitant use of antidepressant and antipsychotic can cause this fata condition and neuroleptic malignant syndrome (NMS). Since there are no laboratory tests available in diagnosing SS, the diagnosis protocol is based on physical examination. There are 3 criteria exited for diagnosing SS, including Sternbach's serotonincriteria (1991), Radomski's revised criteria (2000), and Hunter SS toxicity criteria (2003), which have been listed in Table 1. SS may resolve within 24 hours of onset of symptoms but in severe cases it can be sustained for a long time and leads to death, serious cases may accompany with tachycardia, delirium, hypertonicity, muscular rigidity, and even renal failure. Mild cases may present with tachycardia, mydriasis, diaphoresis, and shivering while moderate may present with hyperreflexia and clonus of the lower extremities and mild agitation,^[10]^ which can be overlooked sometimes. Clinicians may underdiagnose the syndrome due to the lack of certain experience, but it is vital to keep it in mind because it is potentially lethal if not appropriately treated.

**Table 1:**
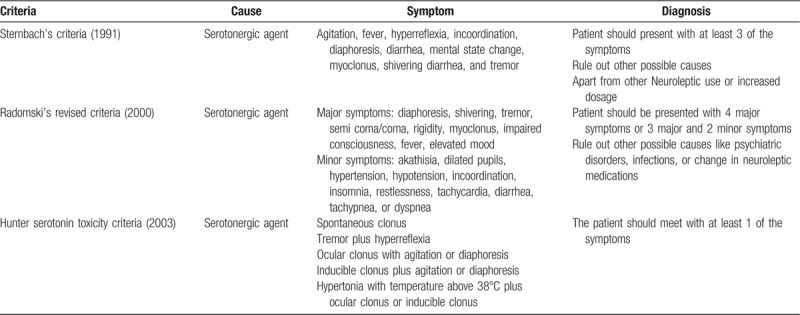
The diagnosis criteria of serotonin syndrome.

The case described here followed an atypical course. The patient was admitted to the hospital for lethargic, less communicative, and insomnia for 20 days. He was taking 30 mg of Paroxetine in local hospital but presented with severe AEs including headache and vomiting. According to regional poison's center it was advised that doses of Paroxetine less than 600 mg were relatively safe and need no treatment.

Thus, he was transferred to our hospital. Considering the diagnosis of “depression,” the patient was administrated with Donepezil (35 mg), Olanzapine (12.5 mg), and Citalopram (80 mg) accordingly. In our case, the presentation of the patient worsened day by day, he was presented with lethargic and shivering first but soon developed to hyperthermia, tachycardia, myoclonusand confusion. We have used Naranjo Algorithm as a tool to evaluate the probability of adverse reaction (ADR) rather than the result of other factors. The score is 4, which indicates a possible ADR. After taking a thorough review of his history including the current medication, physical examination, and exclusion of other causes (encephalitis, septic conditions, and NMS), the diagnosis of SS can be confirmed regarding the presentations of our patient, which include rigidity, shivering, conscious disturbance, hyperthermia, diaphoresis, and tachycardia, which meet all the current 3 diagnosis criteria of SS.^[11–13]^Thus, we stopped all the antidepressants and neuroleptics the patient took and accompanied by other supportive care. Clonazepam was administrated in addition to Cyproheptadine. The symptom resolved after 6 days.

This case had 2 idiosyncratic features. Apart from the fact that the medication the patient took was not in so much dose, he took both serotonergic agents and antipsychotics. Thus, the diagnosis of NMS cannot be excluded. SSRIs are able to trigger SS because of their serotonergic activity that overstimulated the 5-HT1A receptors, which occurred predominantly in the brain. The Citalopram and Paroxetine all belong to SSRIs, but they were considered to be safer in overdose. Citalopram possesses a high selectivity for serotonin reuptake inhibition ^[14,15]^and a weaker CYP450 inhibitory activity^[16,17]^, thus it is considered to be the safest SSRIs. especially when our patient took a normal dose of Citalopram and Paroxetine (up to 30 times the regular daily dose is considered to be moderate overdose which may be associated with minor or no symptoms).^[18]^ However, the patient of our case presented with serious clinical manifestations that included hyperthermia, status epilepticus, coma, muscle rigidity, and autonomic dysfunction. Thus, we have to exclude the possibility of the side effect caused by the combination of other drugs and possibility of NMS. SS and NMS can be overlapping in clinical features like fever, consciousness disturbance, autonomic symptoms, increased creatine phosphokinase levels, and muscular rigidity; we need to differentiate them sometimes.

First, NMS is the AE of antipsychotics while SS is associated with the administration of serotogenic agents. NMS is caused by medications with dopamine blocking properties while the onset of certain symptom is slower and the duration after treatment is longer. NMS can last for several weeks and take 9 to 14 days to remit,^[19]^ while SS can resolve within a few days, the characteristics of these 2 syndromes are summarized in Table 2. Therefore, careful observation of clinical signs and detailed medication history could help us in differentiating these 2 diseases. As for our patient, the medicine that involved includes Olanzapine and Donepezil. Olanzapine is an atypical antipsychotic drug with low potential for extrapyramidal effects and NMS have also been reported in several cases of Olanzapine-induced NMS.^[20,21]^ But the evidence that supports the Olanzapine-induced SS is not enough; Haslett and Kumar have reported a case of Olanzapine-induced SS but was refuted as a misunderstanding of the behavioral serotonin syndrome of rodents and human being, thus the Olanzapine was still to be regarded as unlikely to cause SS.^[22,23]^

**Table 2:**
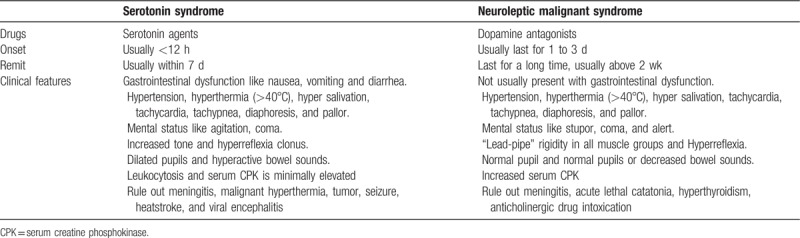
Characteristics of serotonin syndrome and neuroleptic malignant syndrome.

Donepezil is an acetylcholinesterase inhibitor that inhibits the breakdown of acetylcholine and thus increases the availability; it is known to be metabolized mainly by CYP3A4 and to a lesser extent by CYP2D6. Warwick et al^[24]^ have reported the case of a 78-year-old man, who had a pre-existing parkinsonian syndrome who experienced a variant of NMS after the combination use of olanzapine and donepezil . As for our case, the patient has no medical history and is not possible to have NMS after the small dose of olanzapine and donepezil use, considering the medication history and the typical SS, the patient remits after 6 days, thus we exclude the diagnosis of NMS.

Given that the patient was taking a relatively less dose of antidepressant and antipsychotics, could there have been some variation in metabolism of these agents, resulting in increased possibility that led to the subsequent syndrome? Thus, the assumption of the genetic individual vulnerability reported by Direk et al^[25]^ that leads to the high susceptibility of such drugs cannot be excluded. As we know, all the drugs that the patient took are to be metabolized by the cytochrome P450 (CYP) enzyme system. Paroxetine is metabolized by the CYP2D6 system and Citalopram is metabolized by CYP3A4, 2C19, and CYP2D6. Olanzapine is metabolized by CYP2D6, CYP1A2 while donepezil is metabolized mainly by CYP3A4 and CYP2D6. There are 80 variant alleles for CYP2D6 which have been discovered while CYP2D6 gene polymorphisms are also a contributing factor in developing SS and for patients with genetic CYP3A4 variants the excessive level even treated with a normal therapeutic dose was reported too.^[26]^ As to our case, the patient seems susceptible to Paroxetine, since he presented AE immediately after the Paroxetine intake, Paroxetine is reported to be the most potent CYP2D6 inhibitor of all antidepressants ^[27]^ when coadministered with drugs possessing serotonergic activity, the drug levels may increase to cause SS. Thus, the genetic testing was recommended to this patient, but he refused.

In our case, we suppose the sensitivity of SSRIs in our patient may increase serotonin transmission and result in higher serotonin activity ,^[9,28,29]^ because the drug that was used in our patient was not in high dose, thus it is essential for clinicians to keep in mind when patients taking serotonergic agents who demonstrate acute change in their mental status. Besides, clinicians should be aware of such patients who seem to be sensitive to SSRIs, who may require a genetic testing before the initiation of SSRI therapy.

## Author contributions

**Data curation:** Jie Ni.

**Methodology:** Fei He, Yao Liu.

**Supervision:** Hanwen Tong.

**Validation:** Peng Xu.

**Writing – original draft:** Yun Liu.

**Writing – review & editing:** Hailong Yang, Qiulin Zhang, Jun Wang.
